# Harmony

**DOI:** 10.1002/acm2.70154

**Published:** 2025-07-13

**Authors:** Michael D. Mills

**Affiliations:** ^1^ Radiation Oncology University of Louisville Louisville Kentucky USA

What comes to mind when you hear the word *harmonize*? For many in my generation, it may evoke songs like *Bridge Over Troubled Water*, *California Dreaming*, or *Suite: Judy Blue Eyes*—music that transcends its notes to create something that feels deeply right. Harmony can soothe, stir, or even challenge, but at its best, it resonates with purpose and clarity. So too, in a different way, can publishing an academic journal.

My colleague at *Medical Physics*, John Boone, is, I'm told, quite the musician. Though I've never heard him play, our video conferences are always framed by the guitars adorning his wall—a quiet testament to his rock and roll roots. I, too, was once a musician—a trumpet player immersed in the big band era and the brass‐driven sounds of 1970s horn bands. But that was long ago, a different chapter. The trumpet is a demanding companion, and I laid it down more years ago than I care to count. I admire John for keeping music in his life; perhaps we all need something that reminds us of rhythm, timing, and the joy of playing in tune with others.

Which brings me to the central theme of this editorial: harmonizing our two journals—*JACMP* and *Medical Physics*. Why harmonize? The simplest answer is: for the patient. But just as importantly, for you—the author, the reviewer, and the reader. When journals share common standards, processes, and expectations, it becomes easier to collaborate, easier to publish, and easier to transfer manuscripts across platforms. It also helps our publishing partner streamline operations, ultimately improving efficiency and clarity for all stakeholders.

Consider, for example, manuscript structure. What rules should govern the title length? The organization of an abstract? The appropriate formatting of a Research Article versus a Technical Note? While some distinctions may be justified, most are not. Consistency breeds clarity. In that spirit, we are implementing an important change: JACMP will now adopt the structured abstract format used by *Medical Physics*. Beginning immediately, all new submissions must include abstracts organized into five clearly defined sections: Background, Purpose, Methods, Results, and Conclusions. The word limit for abstracts in both journals will now be standardized at 500 words.

1. Structured abstracts are now required for all new *JACMP* Research Article and Technical Note submissions.
2. This format is already well‐established in *Medical Physics*, and most in the *JACMP* community will find it familiar.
3. Education Articles, which may not fit neatly into this structure, will be accommodated with examples to guide your submission.

We encourage all contributors—whether you're preparing a Research Article, Review Article, Technical Note, or Educational manuscript—to review carefully our updated Author Guidelines, as many details have evolved in service of this harmonization effort.

Much like the UK and the USA—nations famously “divided by a common language”—*Medical Physics* and *JACMP* are journals with distinct identities, yet built upon a shared foundation. It's not uncommon to find that a reader regularly engages with one but seldom with the other. After all, keeping up with over 100 articles per month across both is no small feat.

Your focus may lie in mastering a research domain—securing grants, publishing results, and shaping the frontiers of knowledge. Or perhaps your passion lies in clinical excellence—translating knowledge into action to improve directly patient care. Both are vital. Both are heroic in their own ways.

True harmonization is not about sameness. It is about dialogue, respect, and shared rhythm. It's a dance between equals, each bringing their strengths to the floor. As we move toward greater alignment, *JACMP* is making adjustments—trading a bit of historical flexibility for greater narrative precision and structural coherence. But this is not a loss. It is an evolution.

The goal is not to merge or erase differences between journals, but to celebrate those differences more effectively and creatively within a unified publishing framework. From research and imaging to education, oncology, safety, artificial intelligence, radiobiology, and beyond—the diverse cultures within AAPM all deserve a place at the table, a voice in the chorus. The multiple AAPM member cultures require some editorial discretion and independence that must be balanced with harmonization. The editors craft the publication space of our journals; it evolves slightly with each issue, much like jazz performers who never play the same cut in exactly the same way.

There will always be voices that use change as an opportunity to consolidate power. But enlightened leadership listens. It considers the whole. It acts with the patient and the broader membership, in mind. And when governance is truly wise—when the parts come together in harmony—the results can be breathtaking.

Summon the Heroes.

